# Predicting Co-Complexed Protein Pairs from Heterogeneous Data

**DOI:** 10.1371/journal.pcbi.1000054

**Published:** 2008-04-18

**Authors:** Jian Qiu, William Stafford Noble

**Affiliations:** 1 Department of Genome Sciences, University of Washington, Seattle, Washington, United States of America; 2 Department of Computer Science and Engineering, University of Washington, Seattle, Washington, United States of America; Columbia University, United States of America

## Abstract

Proteins do not carry out their functions alone. Instead, they often act by participating in macromolecular complexes and play different functional roles depending on the other members of the complex. It is therefore interesting to identify co-complex relationships. Although protein complexes can be identified in a high-throughput manner by experimental technologies such as affinity purification coupled with mass spectrometry (APMS), these large-scale datasets often suffer from high false positive and false negative rates. Here, we present a computational method that predicts co-complexed protein pair (CCPP) relationships using kernel methods from heterogeneous data sources. We show that a diffusion kernel based on random walks on the full network topology yields good performance in predicting CCPPs from protein interaction networks. In the setting of direct ranking, a diffusion kernel performs much better than the mutual clustering coefficient. In the setting of SVM classifiers, a diffusion kernel performs much better than a linear kernel. We also show that combination of complementary information improves the performance of our CCPP recognizer. A summation of three diffusion kernels based on two-hybrid, APMS, and genetic interaction networks and three sequence kernels achieves better performance than the sequence kernels or diffusion kernels alone. Inclusion of additional features achieves a still better ROC_50_ of 0.937. Assuming a negative-to-positive ratio of 600∶1, the final classifier achieves 89.3% coverage at an estimated false discovery rate of 10%. Finally, we applied our prediction method to two recently described APMS datasets. We find that our predicted positives are highly enriched with CCPPs that are identified by both datasets, suggesting that our method successfully identifies true CCPPs. An SVM classifier trained from heterogeneous data sources provides accurate predictions of CCPPs in yeast. This computational method thereby provides an inexpensive method for identifying protein complexes that extends and complements high-throughput experimental data.

## Author Summary

Many proteins perform their jobs as part of multi-protein units called complexes, and several technologies exist to identify these complexes and their components with varying precision and throughput. In this work, we describe and apply a computational framework for combining a variety of experimental data to identify pairs of yeast proteins that partipicate in a complex—so-called co-complexed protein pairs (CCPPs). The method uses machine learning to generalize from well-characterized CCPPs, making predictions of novel CCPPs on the basis of sequence similarity, tandem affinity mass spectrometry data, yeast two-hybrid data, genetic interactions, microarray expression data, ChIP-chip assays, and colocalization by fluorescence microscopy. The resulting model accurately summarizes this heterogeneous body of data: in a cross-validated test, the model achieves an estimated coverage of 89% at a false discovery rate of 10%. The final collection of predicted CCPPs is available as a public resource. These predictions, as well as the general methodology described here, provide a valuable summary of diverse yeast interaction data and generate quantitative, testable hypotheses about novel CCPPs.

## Introduction

Proteins carry out most of the work in the cell, and they frequently do so by interacting with other proteins. Therefore, understanding protein and hence cellular function often entails knowing about various types of protein-protein interactions. This paper describes a method for predicting these interactions using a supervised classification algorithm that learns from a variety of genome-wide data sets.

Three classes of methods for predicting protein-protein interactions are described in the scientific literature. The first class consists of docking methods that employ detailed molecular simulations to dock two protein structures. These methods do not scale to the entire genome, both because they require protein structures and because they are computationally expensive. High-throughput computational methods fall into two classes: those that predict direct physical interactions [1]–[9], and those that predict both direct and indirect interactions (i.e., co-membership in a protein complex) [10]–[13]. The current work focuses on the latter problem: predicting co-complexed protein pairs (CCPPs).

We frame the problem as a supervised learning problem, and we train a support vector machine (SVM) classifier to discriminate between pairs of proteins that are co-complexed and pairs that are not. The SVM is a non-parametric statistical method for discriminating between two classes of data. SVMs have been applied widely in bioinformatics, in applications as diverse as protein homology detection, alternative splicing prediction, microarray analysis and mass spectrometry analysis [14]. Most relevantly, they have been used successfully to recognize physically interacting pairs of proteins [3],[5],[9]. The SVM operates by projecting the data into a vector space and finding a line (or, more generally, a hyperplane) that separates the classes in that space. SVMs are motivated by statistical learning theory, which suggests an optimal method for identifying this separating hyperplane. Furthermore, SVMs are part of a class of methods, known as *kernel methods*, that make use of a specific notion of pairwise similarity (*kernel functions*) to project data into a high-dimensional vector space. The benefits of the kernel approach are three-fold: the kernel function can incorporate prior knowledge of the problem domain; the kernel function can operate on non-vector data such as strings, sets or graphs, and kernel algebra allows us to combine heterogeneous types of data within a single classification framework. The SVM algorithm and its application to biological data is described in an accessible fashion in [15]; a much more detailed description of SVM applications in computational biology is available in [16].

The ability to learn from heterogeneous data is of particular value in the prediction of CCPPs, because so many types of data are relevant to this task. In this work, we define separate kernels that operate on each relevant data type. These include three kernels on protein sequences, three kernels on different types of protein networks derived from high-throughput data, and kernels on gene expression, interologs, Gene Ontology terms, co-regulation and localization data. We combine all of these kernels in a single classifier that achieves state-of-the-art predictive accuracy.

In this work, we demonstrate the utility of a particular type of kernel, the *diffusion kernel* [17], for predicting CCPPs. The diffusion kernel can be naturally applied to protein interaction networks. Various types of networks, representing protein physical interactions, complexes and genetic interactions, can be identified by large-scale experiments: yeast two-hybrid assays for physical interaction detection [18],[19], affinity purification coupled with mass spectrometry (APMS) for complex detection [20],[21],[22], and large-scale mapping of genetic interactions [23].

The resulting protein interaction networks have been shown to exhibit several distinctive properties [11],[23]. First, the degrees of vertices exhibit a power-law distribution, with many vertices having a small number of connections, and few vertices having a large number of connections. Second, the networks belong to the class of small world networks and contain densely connected local neighborhoods.

These network properties can be exploited to improve statistical inferences about protein-protein interactions. Tong et al. [23] showed that, although there is small overlap between genetically interacting protein pairs and CCPPs, proteins sharing a large number of neighbors in the genetic interaction network tend to be members of the same complex. Goldberg and Roth [11] used a mutual clustering coefficient (MCC) to describe the cohesiveness in the physical interaction network. They showed that vertices with a high MCC are more likely to share an edge and that ranking by MCC improves the accuracy of edge inference.

MCC considers the number of common neighbors shared by two vertices, i.e., it only considers paths of length two. In this study, we generalize upon MCC by using the diffusion kernel, which takes into account paths of all lengths [17]. The diffusion kernel quantifies the distance between two nodes as the weighted sum of all paths connecting them, assigning larger weights to shorter paths. Our experiments show that the diffusion kernel performs much better than MCC in ranking protein pairs. In addition, we show that using a diffusion kernel in the context of an SVM classifier improves upon direct ranking by the diffusion kernel alone, and that a diffusion kernel performs much better than a linear kernel, which also only considers paths of length 2 in a network.

Next, we show that integration of different data sources improves the performance of our classifier. The summation of sequence and diffusion kernels yields much better performance than either sequence or diffusion kernel alone. This classifier successfully identifies 4789 out of 10980 positives before producing the first false positive. The addition of features such as co-expression, co-regulation, interolog, co-localization and GO annotation improve the ROC_50_ score from 0.859 to 0.937.

We also validate our method using two recently decribed large scale APMS data sets [21],[22]. When these data sets are not used for training, our predicted positives are significantly enriched with protein pairs that occur in both data sets.

After validating our method, we trained two SVM classifiers, one using all available data, and one that excludes GO annotations, and applied both classifiers to all pairs of yeast proteins. The resulting predictions are available through the Yeast Resource Center (http://www.yeastrc.org/pdr) [24].

## Methods

### Gold Standard Protein Pairs

We derive the labels for our classification task from the MIPS complex catalogue version 18052006 [25] excluding category 550: complexes by systematic analysis. The rest of the MIPS complex catalogue contains manually curated complexes derived from the scientific literature. This manually curated database is believed to be highly accurate and has been used to define gold standard CCPPs in several studies [5],[10],[12],[26]. The MIPS complex catalogue organizes complexes into a hierarchy, with each lower level sub-complex contained within the corresponding upper level complex. Our CCPPs come from the lowest level and are hence the most specific complexes in the MIPS complex catalogue. The set consists of 217 complexes, containing 1190 proteins and 10,980 CCPPs.

We select negative examples (non-CCPPs) at random from among all protein pairs that do not co-occur in any top level MIPS complex [9],[12],[26]. The resulting set of negatives may be contaminated with some positive CCPPs; however, given the ratio of co-complexed versus non-co-complexed pairs in the yeast genome, the level of contamination is likely to be low. Several studies [10],[27] have attempted to remove these false negative CCPPs from the gold standard by requiring that non-CCPPs localize to different cellular compartments. However, Ben-hur and Noble [28] have shown that this strategy constrains the distribution of negative examples in such a way that the classification task becomes significantly easier. We use a data set with the number of negative examples the same as positive examples to compare the performance of various methods, and use a larger data set with a negative-to-positive ratio of 10 to estimate the false discovery rate.

The complete collection of labeled examples, as well as all of the kernels described in the next section, are available at the on-line supplement http://noble.gs.washington.edu/proj/coco.

### Kernel Methods

A kernel method is an algorithm that can be written such that all occurrences of data vectors appear within a scalar product operation. When this is the case, the scalar product operation <*X_i_*,*X_j_*> can be replaced with a generalized similarity function *K*(*X_i_*,*X_j_*), known as the *kernel function*. If the kernel function is positive semidefinite and symmetric, then there provably exists some vector space (the *feature space*) in which the kernel function plays the role of the scalar product. In other words, if Φ defines a mapping from the space that the data resides in (the *data space*) into the feature space, then Φ(*X_i_*)×Φ(*X_j_*) = *K*(*X_i_*,*X_j_*). The kernel function provides an intuitive way to encode prior knowledge about a data set. Furthermore, kernel methods provide a natural way of combining heterogeneous data sources [29],[30], because the sum of two kernels is itself a kernel and is equivalent to concatenating the vector representations of each data point in the two corresponding feature spaces. This capability is particularly valuable in the context of predicting CCPPs, because so many types of data are relevant.

Predicting edges in a protein interaction or co-complex network presents an additional difficulty for which kernels can provide a solution. Many relevant types of data—protein sequence, gene expression, etc.—concern individual proteins, whereas the predictor evaluates protein pairs. This begs the question, how do we define a similarity between two pairs of proteins, given a similarity function that is defined on single pairs. Several groups have used SVMs to predict protein-protein interactions [5],[9] and have used a tensor product transformation to derive a kernel on protein pairs from a kernel on individual proteins. Given a kernel *K* that measures the similarity between two proteins, the corresponding tensor product pair kernel (TPPK) *K_p_* is defined as *K_p_* ((*A*,*B*),(*C*,*D*)) = *K*(*A*,*C*)*K*(*B*,*D*)+*K*(*A*,*D*)*K*(*B*,*C*). It is straightforward to show that the feature space of *K_p_* defined on protein pairs is equivalent to the tensor product of the feature vector spaces of *K* defined on individual proteins.

In this work, we employ a variety of kernel functions. Three different amino acid sequence kernels are described in the next section. For several vector data types, we use a radial basis kernel (RBF) *K_R_*(*A*,*B*) = *exp*(−γ∥*A*–*B*∥*^2^*), with γ = 0.5. Finally, for networks, we use the diffusion kernel, defined as follows. Given a graph *G* = (*V*,*E*), define a generator matrix H:
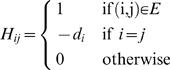
The generator matrix *H* corresponds to the adjacency matrix with the diagonal entry equal to the negative of the degree of the corresponding vertex. The diffusion kernel matrix *K_D_* is then computed as the exponential of the generator matrix: *K_D_* = *e* ^β*H*^. *K_D_*(*i*,*j*) can be regarded as the sum of probabilities of reaching *j* from *i* following all paths from *i* to *j* in a random walk. The parameter β controls how rapidly a random walk diffuses away from a vertex. In this study, we use a fixed diffusion parameter of 1. It can be shown that the exponential of any symmetric matrix is symmetric and positive semidefinite. Therefore, the matrix *K_D_* is a kernel matrix.

### Data Types

Our CCPP predictor combines eleven different data types, listed in Table 1. We first describe six data types that describe individual proteins, followed by five data types that describe protein pairs.

**Table 1:** Data types used in this study.

Data type	Kernel	Type	Number
Protein sequence	Pfam	Protein	6700
Protein sequence	motif	Protein	6622
Protein sequence	spectrum	Protein	6700
Yeast two-hybrid	diffusion	Protein	4155
Genetic interaction	diffusion	Protein	3344
Affinity capture-MS	diffusion	Protein	3627
Interologs	RBF	Pair	1615
Co-expression	RBF	Pair	6320
Gene ontology	RBF	Pair	5306
Co-regulation	RBF	Pair	6270
Co-localization	RBF	Pair	4156

Some types of data described individual proteins and are transformed to pairs by using the TPPK. The remaining data types naturally describe pairs of proteins and can be used directly. The last column indicates the number of proteins with information available for a particular type of data.

### Individual proteins

#### Sequence kernels

We include three sequence-based kernels in this study: the spectrum kernel [31], the motif kernel [32] and the Pfam kernel [3]. The spectrum kernel maps a protein to the space of all subsequences of a fixed length (3-mers, in this case). The feature vector of a protein contains the number of times each 3-mer occurs in its sequence. The motif kernel relies on a pre-defined database of motifs, and represents each protein by the number of times each sequence motif is present in its sequence. The sequence motifs are derived from the eMotif database [33]. Pfam is a database of protein domain families represented as hidden Markov models (HMMs) [34]. Each protein sequence is compared against every HMM in Pfam, and the E-value statistic is computed. The Pfam kernel then describes a protein as a vector of log E-values of such comparisons.

#### Diffusion kernels

We apply the diffusion kernel to three kinds of networks derived from the BIOGRID database [35]. The networks consist of data generated by using (1) yeast two-hybrid, (2) affinity capture-MS and (3) genetic interactions, including synthetic lethality, synthetic growth defect, synthetic rescue, dosage rescue, dosage lethality, dosage growth defect and epistatic miniarray profile.

### Protein pairs

For the remaining data sets, rather than define a kernel on proteins and then apply TPPK, we directly compute features of protein pairs. We then concatenate the resulting features and apply a radial basis kernel to the resulting vectors.

#### Co-expression

Two proteins in the same complex are likely to exhibit correlated gene expression profiles. We derive co-expression features from five different microarray expression data sets: diauxic shift [36], sporulation [37], cell cycle [38], environment [39] and deletions [40]. A scalar product kernel is computed for each expression data set after centering and normalization. The feature value of a protein pair is the computed kernel value of the two proteins in the pair. Each expression data set is treated as one feature. Missing values are replaced with the median values for the remaining protein if one protein in the pair is missing or with the median values of all values if neither protein has observed data.

#### Co-regulation

Proteins in the same complex are likely to be under similar transcriptional control. The same transcriptional regulator might bind to their regulatory elements, which can be measured by ChIP-chip assays. We compute the co-regulation kernel based on the data from Lee et al. [41] This data set contains the binding strengths of 113 transcription regulators to the DNA elements upstream of their regulated genes. For a regulator, the feature value of a protein pair is computed as the lower binding strength of the two corresponding gene regulatory regions by the regulator. The binding strength of a regulator to a gene regulatory element is the negative logarithm of the *p* value reported by Lee et al. [41] The co-regulation data for each regulator is treated as a different feature.

#### Interologs

If the homologs of two proteins in another organism are in the same complex, then these two proteins are also likely in the same complex in yeast [42]. We use PSI-BLAST [43] to identify sequence homologs. All complexes in BIND [44] not in *Saccharomyces cerevisiae* are used to infer the CCPP relationships. All the yeast ORF sequences are searched against the non-redundant database for two iterations with an E-value threshold of 0.005 to generate a position-specific scoring matrix. The matrix is then used to score the yeast ORFs against a database consisting of proteins in non-yeast BIND complexes for one iteration with an E-value threshold of 10 to identify the homologs. The feature values based on interologs can then be computed based on the negative log E-values between sequences: *h*(*A*,*B*) = *max_i_*_,*j*_{*I*(*i*,*j*)*min*(*l*(*A*,*i*),*l*(*B*,*j*))}, where proteins *i* and *j* are sequence homologs of *A* and *B* respectively, *I*(*i*,*j*) is an indicator function indicating that *i* and *j* are two proteins in the same complex from another organism based on BIND, and *l*(*A*,*i*) is the negative log E-value from PSI-BLAST search between protein *A* and protein *i*.

#### Co-localization

If two proteins are in the same complex, then they must be localized to the same compartment at some time. Fluorescence microscopic studies have been used to map the location of yeast proteins on a genomic scale. We derive a co-localization kernel based on the results of Huh et al. [45] Let *f_l_* be the fraction of proteins present in a location *l*, and let *I*(*i*,*l*)be an indicator function indicating that protein *i* is observed to localize to *l*. The co-localization feature values are then computed as follows: *l*(*A*,*B*) = *max_l_*{*I*(*A*,*l*)*I*(*B*,*l*)(−*log*(*f_l_*))}; i.e., the feature value for protein pair (*A*,*B*) is computed as the negative logarithm of the fraction of proteins present in the most specific location where both proteins *A* and *B* are observed to localize.

#### Gene Ontology terms

The Gene Ontology (GO) [46] is a collection of standardized terms to describe the molecular function, biological process or cellular component in which a protein participates. If a protein is annotated with a GO term *T*, then we also add the annotations of all ancestors of *T* to the protein. Two proteins in the same complex are more likely to have similar GO term annotations. Let *f_g_* be the fraction of proteins annotated with a particular GO term *g*, and let *I*(*i*,*g*) be an indicator function indicating that protein *i* is annotated with GO term *g*. The GO feature values are then computed as follows: *G*(*A*,*B*) = *max_g_*{*I*(*A*,*g*)*I*(*B*,*g*)(−*log*(*f_g_*))}; i.e., a single feature value for protein pair (*A*,*B*) is computed as the negative logarithm of the fraction of proteins present in the most specific GO term where both proteins A and B have annotations. We derive three different features for the three ontologies.

### Combining all of the kernels

The eleven different kernels are combined in two stages. First, the three sequence kernels and the three diffusion kernels are individually normalized by projecting onto the unit sphere, via 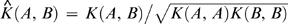. The six kernels are then summed in an unweighted fashion. The TPPK transformation is applied to this summed kernel, and the result is added to the RBF kernel defined on the five pairwise data types. With some abuse of notation, the final kernel can be represented as follows:
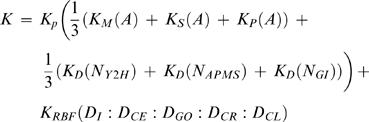
where *A* represents the amino acid sequences, the three *N*'s represent the three interaction networks, the five *D*'s represent five pairwise data types, and “:” indicates vector concatenation.

### Experimental Framework

We use the publicly available PyML implementation of the support vector machine algorithm (http://pyml.sourceforge.net). Three-fold cross-validation with *C* = 10 is carried out to evaluate the performance. In each split, each partition contains the same number of positive and negative data points.

We measure the quality of a CCPP classifier by using receiver operating characteristic (ROC) curves. This curve plots number of true positives as a function of number of false positives for varying classification thresholds. Our performance metric is ROC_50_, the normalized area under this curve, up to the 50th false positive. A perfect classifier receives an ROC_50_ score of 1.0; a random classifier receives a score close to 0.

The ROC curve does not take into account the negative-to-positive ratio in the data set. In the application of a classifier, we are often interested in the false discovery rate, the fraction of predicted positives that are false positives. This metric is highly dependent on the negative-to-positive ratio. We report the false discovery rate of the kernel with all features assuming a negative-to-positive ratio of 600, which is the estimated ratio in the real scenario [26].

## Results

### Diffusion Kernels Yield Superior Performance

**Figure 1:**
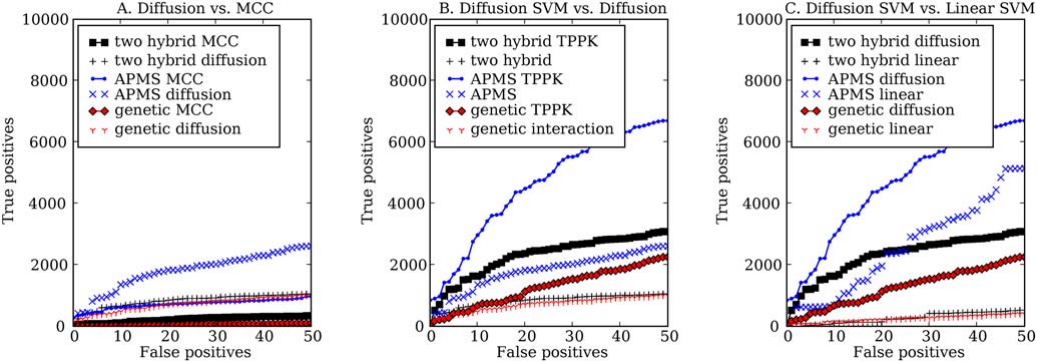
The ROC_50_ performance of the TPPK of diffusion kernels. (A) compares the ROC_50_ scores from ranking directly based on diffusion kernel values and from ranking on hypergeometric MCCs. The ROC_50_ scores of the TPPK of diffusion kernels are compared with (B) ranking directly on the diffusion kernel values and (C) the TPPK of linear kernels.

We begin by demonstrating that, when directly ranking protein pairs, the diffusion kernel improves the quality of the CCPP predictor. Goldberg and Roth [11] introduced the hypergeometric mutual clustering coefficient (MCC) and showed that it had the best performance among four MCC formulations in ranking high confidence protein-protein interaction edges above low confidence ones. The MCC only considers paths of length two in a network. The diffusion kernel, on the other hand, considers paths of all lengths connecting two proteins. We compare ranking based on the diffusion kernel values with ranking by the hypergeometric MCC. The results in Figure 1A show that the diffusion kernel produces a better ranking than the hypergeometric MCC for three different types of networks—yeast two-hybrid, APMS and genetic interactions. This result demonstrates that taking into account paths of all lengths with the diffusion kernel improves edge inference accuracy.

Next, we show that using a supervised learning algorithm improves over direct ranking. We train a support vector machine using the TPPK of the diffusion kernel, and we compare the SVM's performance with that of the simple method of ranking pairs by the diffusion kernel values between the two vertices directly. Figure 1B shows that, for all three types of networks, the SVM classifier performs better. Among the three networks, APMS yields the best performance. This is not surprising because we are predicting CCPPs, which are directly measured by APMS.

Finally, we show that, in the context of SVM classification, the diffusion kernel yields better performance than the simple linear kernel. Figure 1C compares the ROC_50_ plots of SVMs trained using the same networks but two different kernels. To compute the linear kernel, each row of the adjacency matrix of a network is treated as a feature vector, and the inner products between two rows are computed as the corresponding kernel value. Like MCC, the linear kernels consider only paths of length two. We normalize the linear kernels and transform them using TPPK, as was done for diffusion kernels. Figure 1C shows that, in the setting of SVM classifiers, the diffusion kernels perform much better than the linear kernels for all three networks.

### Combining Kernels from Heterogeneous Data Improves Performance

**Figure 2:**
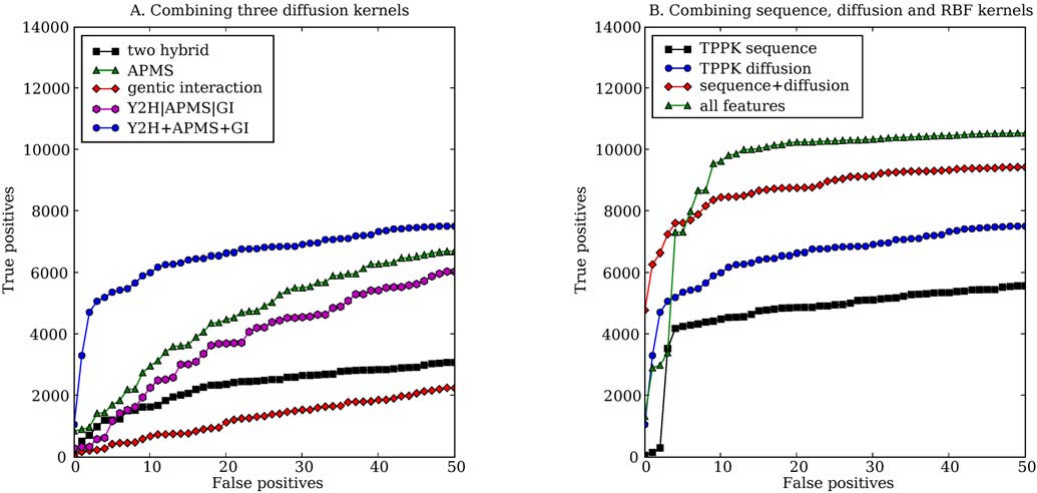
Combining different kernels. (A) Two-hybrid, APMS, and genetic interaction represent the performance of an SVM trained using the TPPK of the diffusion kernel on a single network. Y2H|APMS|GI uses the diffusion kernel derived from a single network containing two-hybrid, APMS, and genetic interaction edges. Y2H+APMS+GI represents the summation of three diffusion kernels based on the three networks. (B) The TPPK sequence kernel is the TPPK of the summation of Pfam, motif, and spectrum kernels. The TPPK diffusion kernel is the same as Y2H+APMS+GI in (A). The sequence+diffusion kernel is the TPPK of the summation of the three sequence kernels and the three diffusion kernels. The all features kernel is the summation of the sequence+diffusion kernel with the RBF kernel.

Different types of high-throughput assays yield complementary information about CCPPs. We therefore trained a single SVM using all three networks simultaneously. Figure 2A shows the results of combining the three networks. We consider two ways to combine the networks: combining adjacency matrices and then performing the diffusion, versus performing diffusions separately on each network and then summing the kernels. Our result indicates that the second approach works better in predicting CCPPs. Combining the three networks into one network fails to preserve the different semantics associated with the three types of edges, leading to worse prediction performance. Indeed, combining the three networks in this fashion leads to even worse performance than is given by the best single network (APMS). In contrast, diffusing on each network separately and subsequently summing the three diffusion kernels improves significantly over the APMS diffusion kernel alone.

Finally, we combined the diffusion kernels with the sequence kernels and with the five protein pair data sets. As shown in Figure 2B, the combination of the diffusion kernels with the sequence kernels perform better than both the summation of the three diffusion kernels and the summation of the three sequence kernels. In particular, in a cross-validated test, the sequence and diffusion kernel is able to rank 4789 out of 10980 positives above all negatives. The addition of the RBF kernel based on co-expression, interolog, co-regulation, co-localization and GO annotation features further improves the ROC_50_ performance from 0.859 to 0.937.

The accuracy of the top-ranked predictions with the inclusion of additional RBF kernel seems to decrease compared with the TPPK of sequence and diffusion kernels alone, as indicated in the leftmost region of the ROC_50_ plot. In particular, six pairs are ranked high by the final classifier but are not labeled as positives according to MIPS. We investigated each of these pairs. The proteins ARC40 and ARC35 are annotated to be in complex ARp2/3 complex by the Saccharomyces Genome Database (SGD), and NOP14 and UTP7 are annotated to be in complex U3snoRNP by SGD. Thus, these two pairs are likely true positives missed by the MIPS database. In another top-ranked pair, UTP9 is a component of the U3snoRNP that is involved in processing of pre-18S rRNA, and CBF5 is the pseudouridine synthase catalytic subunit of box H/ACA snoRNPs, which is also involved in rRNA processing. This pair has been identified by two APMS studies [22],[47]. The classifier with both the RBF kernel and TPPK of sequence and diffusion kernels also predicts PDA1 and KGD1 to be in the same complex with high confidence. PDA1 is the E1 alpha subunit of the pyruvate dehydrogenase complex, and KGD1 is a component of the mitochondrial alpha-ketoglutarate dehydrogenase complex. Both proteins bind to mitochondrial DNA and are part of mitochondrial nucleoid. Finally, for two pairs, this classifier predicts one protein in the kinetochore, DAD2 or DAD4, to be in the same complex with one protein in the spindle pole body, CNM67 or SPC98. Although the kinetochore and the spindle pole body are both part of spindle, they are two separate components. The classifier has difficulty distinguishing these two components from each other. Thus the apparently worse performance of the classifier with the additional RBF kernel in the leftmost region of the ROC plot may partially be due to the presence of true CCPPs in the negative training set as a result of incomplete MIPS annotations.

### The SVM Makes Predictions from Partial and Indirect Evidence

A significant concern for any method that simultaneously exploits multiple types of data arises from the increased prevalence of missing data. If each given data type is missing 10% of its entries, then in a data set consisting of four such data sources the probability that a given example will have missing data from at least one source is 1−0.9^5^ = 41.0%. In our experiments, most of the data sources have a significant proportion of missing data, and the coverage varies across the data sets. Table 1 lists the number of proteins with information available for each type of data used in this study. Not surprisingly, the sequence kernels have the highest coverage, whereas the interolog feature and the three networks have the lowest coverage.

**Figure 3:**
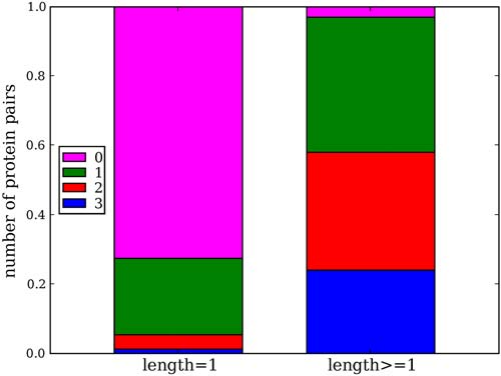
Prediction of CCPPs does not require the availability of data in all three networks. In the figure, we examine the set of 7510 correctly predicted interactions before the 50th false positive for an SVM trained using all three diffusion kernels. The figure plots the percentage of correctly predicted protein pairs that are directly linked (“length = 1”) or linked by a path of any length (length≥1) in three, two, one or none of the three interaction networks.

We therefore investigated how much the SVM's performance depends on the availability of all the data sources used. We examined the 7,510 true positive interactions identified before the 50th false positive for the SVM trained using the summation of the three diffusion kernels. For each correctly predicted protein pair and each of the three networks, we asked whether there exists an edge between the proteins and whether there exists a path of any length between the two proteins. Figure 3 shows that most of the correctly predicted pairs are not directly linked in any of the three networks. Indeed, only a relatively small percentage of the protein pairs are linked by a path of any length in all three networks. These results demonstrate that the SVM is capable of making correction predictions from partial and indirect evidence.

### Estimation of the False Discovery Rate

In the previous sections, we compared the performance of various methods using a data set with the number of negatives chosen to be the same as the number of positives. In reality, the number of negatives is much larger than that of positives, and the negative-to-positive ratio has been estimated to be around 600 [26]. We next estimate the false discovery rate of the classifier with both the RBF kernel and the TPPK kernel assuming a negative-to-positive ratio of 600. Ideally, we would like to train on a training set with the number of negatives equal to 600 times the number of gold standard positives in our data set. However, this would involve training an SVM on a data set of 10,980×601 = 6,598,980 protein pairs, which is not computationally feasible. Therefore, we instead perform three-fold cross validation on a data set with the number of negatives chosen to be ten times the number of positives. In the computation of the false discovery rate, each occurrence of a false positive is then multiplied by 60 to simulate a negative-to-positive ratio of 600.

We train two classifiers, one that uses GO term annotations and one that does not. GO term annotations are sometimes derived from experimental observations of physical interactions or complex memberships. Using GO term features may therefore artificially inflate the performance of the classifier. To eliminate the possibility of circularity, we trained a classifier using the TPPK kernel and the RBF kernel without GO term features. On the other hand, biologists may be interested in the best possible predictions we can get using all available data. Especially when we apply the classifier to the prediction of protein pairs without interaction data, the issue of circularity is not a concern, and GO term annotations from sources other than physical interaction or complex membership may be useful in CCPP prediction. Therefore, we also trained a classifier that includes the GO term features.

**Figure 4:**
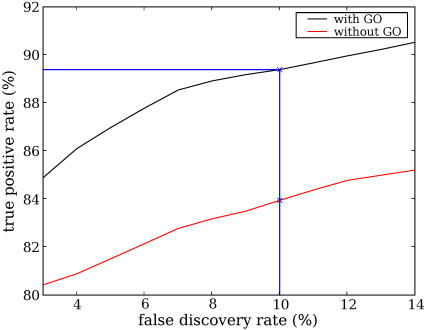
The true positive rate vs. false discovery rate of two classifiers using both the RBF and TPPK kernels. The false discovery rate (FDR) was estimated assuming a negative-to-positive ratio of 600∶1. The black line indicates the performance of the classifier using all features, while the red line indicates the performance of the classifier without using the features based on GO term annotations. The *x*-axis begins at an FDR of 3% because very small FDRs cannot be estimated accurately for this dataset.

Figure 4 plots the true postive rate (TPR) as a function of the false discovery rate (FDR) for our classifiers with and without using GO term features. To make this plot, we estimate the false discovery rate separately for each fold of the cross validation with the following formula:
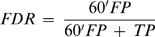
FP and TP are the number of false and true positives for a certain threshold, respectively. For a certain number of false positives, there often exist multiple corresponding numbers of true positives. The median TP is used to compute a discrete sequence of observed FDR and TPR. Linear interpolation is then used to compute the TPR for all FDR values between any two adjacent observed FDRs. The average TPR across the three folds for a certain FDR is reported in Figure 4. Not surprisingly, inclusion of the GO term features improves the performance. With a false discovery rate of 10%, the classifier without using GO term features achieves a true positive rate of 83.9% and the classifier using GO term features achieves a true positive rate of 89.3%.

### Validation of the APMS Data

**Figure 5:**
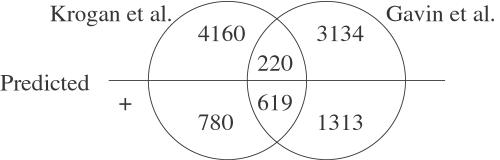
Enrichment of reliable pairs in predicted positives. The figure shows the number of protein pairs in the APMS datasets of Krogan et al. and Gavin et al. that are predicted to be negative (above the line) or positive (below the line) by our approach. Pairs from the MIPS training set are excluded. The predicted positive set is enriched with reliable pairs that are identified by both groups.

Advances in tandem affinity purification (TAP) followed by mass spectrometry make it possible to characterize complexes on a large scale. Recently, two groups published high throughput identifications of complexes in *Saccharomyces cerevisiae* [22],[21]. Krogan et al. [22] identified 7,076 CCPPs, and Gavin et al. [21] identified 6,531 CCPPs. However, these two data sets have only 1,542 CCPPs in common. We used our MIPS training set to train a model based on all our features with these two data sets excluded from the APMS diffusion kernel. We then applied the trained model to the prediction of CCPPs among pairs identified by Krogan et al. or Gavin et al. Our model predicted 4536 pairs to be positive, including 1824 pairs present in the training set and 2712 new predictions. Figure 5 shows the number of pairs in the intersections between the two APMS data sets and our predicted positive data set after removal of pairs in the MIPS training set. Among the 2712 predicted positive pairs, 619 (22.8%) pairs are present in both APMS data sets. This ratio is much higher than that in the whole data set (8.2%). Given that the number of pairs in either APMS data set is 10,226, and the number of pairs in both APMS data sets is 839, if we randomly pick a subset of 2712 pairs, the Fisher exact test *p*-value of the subset containing at least 619 pairs in both APMS data sets is 4.2e–198. Because the pairs in both APMS data sets are believed to be more reliable than the rest of the pairs in the data set, it is reassuring that the positives predicted by our model are enriched in these reliable pairs. Our model predicted a larger fraction of pairs to be positive in the data set of Gavin et al. (48.5%) than in the data set of Krogan et al. (38.0%)

Collins et al. [48] recently developed a Purification Enrichment score and used this score to combine the two APMS data sets and generate a data set of high accuracy. We compared the overlap between our predicted positives and the data set of Collins et al. Among the 10,226 pairs in either APMS data set but not in our training set, 2985 pairs are present in the data set of Collins et al. The 2712 pairs predicted to be positive by our classifier contain 1882 of the 2985 pairs. This large degree of overlap between our predicted positives and the data set of Collins et al. is statisitically significant according to Fisher's exact test (*p*<1*e*–300). Thus, the CCPPs predicted by our classifier are consistent with the results of Collins et al.

### Comparison with Related Work

Qi et al. [26] recently performed an extensive study comparing multiple methods on the prediction of complex co-memberships, physical interactions and co-pathway relationships. The study concludes that, among various classification algorithms, random forests performs the best, with random forest-based k-nearest neighbor and SVMs following closely. We applied our kernel methods to their gold standard data set following their learning procedure. 30,000 protein pairs were randomly picked as the training set with 50 from the positive data set and 29,950 from protein pairs not in the positive data set. Another 30,000 protein pairs were picked randomly from the remaining protein pairs as the test set. The test set also contained 50 pairs randomly picked from the positive data set. This training and testing procedure was repeated 5 times instead of 25 times as done by Qi et al. to save time. Our approach with both the RBF and TPPK kernels has a mean ROC_50_ of 0.69 with standard deviation of 0.05. This is slightly better than the best result (0.68) by Qi et al. Qi et al. published their study before the availability of the two recent large scale APMS studies [21],[22]. We removed these two data sets from the APMS network and tested on the data set of Qi et al. The mean ROC_50_ is 0.68 with a standard deviation of 0.05. This is similar to what Qi et al. reported as their best performance.

Qi et al. simulates a realistic scenario by using a negative-to-positive ratio of 600∶1 in the training set. However, in their setting, each classifier only learns from 50 positive pairs. Because of this relatively small number of positives in the training set, the resulting classifier will likely not generalize as well as a method that learns from all available positive pairs. This is why we instead chose to train on a data set with all available positive training pairs and a negative-to-positive ratio of 10, and simulate the real scenario by magnifying each false positive by 60, as described above.

### Predictions for All Yeast Protein Pairs

**Figure 6:**
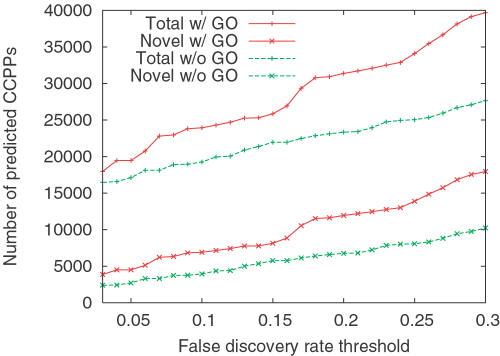
Total number of predicted CCPPs as a function of FDR threshold. The red and green series correspond to classifiers trained with and without GO term annotations, respectively. The upper two series (marked with “+” signs) represent the total number of predictions, and the lower two series (marked with x's) represent the number of novel predictions (i.e., excluding protein pairs in the MIPS complex catalogue).

Having demonstrated that our method produces accurate predictions, we proceeded to apply the two classifiers described previously—trained with and without GO annotations—to all protein pairs in *Saccharomyces cerevisiae*, excluding 809 dubious open reading frames and 7 pseudogenes. For the SVM trained without GO term annotations, 19,258 out of 17,307,786 protein pairs are identified using an FDR threshold of 10%, including 3,946 pairs that are not already annotated in the MIPS complex catalogue. Figure 6 shows the number of predicted pairs as a function of FDR threshold for both classifiers. As expected, at a given FDR threshold, the classifier trained with GO terms predicts more protein pairs than the classifier trained without GO terms. Both sets of predictions can be downloaded from Yeast Resource Center Public Data Repository (http://www.yeastrc.org/pdr), and all predictions obtained using an FDR threshold of 10% are included in the browseable interface of the repository.

We analyzed the novel predictions produced by the classifier trained without GO annotations. First, we divided these novel predictions into two sets: those protein pairs in which one protein is a member of a MIPS complex, and those pairs in which neither protein is in the manually curated MIPS complex catalogue. Among the 3,946 novel CCPPs, 3,260 pairs are linked to one of the 1,237 members of any MIPS complex, and the remaining 686 pairs do not involve any MIPS complex.

We began by investigating the extent to which the former set of predictions extend known MIPS complexes. Ideally, a newly identified member of a protein complex would be predicted to co-complex with all known members of that complex. We therefore identified all proteins that are predicted to be co-complexed with every member of a known MIPS complex containing at least five proteins. These predictions are listed in Table 2. Not surprisingly, the majority of these predictions are not truly novel; rather, they reflect the incompleteness of the MIPS annotation that we used to train our SVMs. In fact, for all predicted new members in Table 2, we were able to find convincing evidence in the scientific literature supporting the prediction , and the citations are given in the table. All the six predicted new members of the mRNA splicing complex—SMB1, SNU114, SYF2, CLF1, ISY1 and CUS1—have GO annotations of “nuclear mRNA splicing, via spliceosome.” The predicted new member of the exocyst complex 160, EXO84 has the GO annotation “exocyst.” SOH1 (MED31), the predicted new member of the mediator complex has been annotated by SGD to be part of the mediator complex. Finally, our classifier also predicted MHR1 to be part of mitochondrial ribosomal large subunit. Although MHR1 is primarily annotated to be involved in homologous DNA recombination and genome maintenance in mitochondria, Gan et al. [49] has shown that MHR1 is present in the mitochondrial ribosomal large subunit fraction separated by sucrose density gradient centrifugation, and the stoichiometry of MHR1 in purified large subunit is roughly equal to that of MRPL1, a member of mitochondrial ribosomal large subunit. In addition, Gavin et al. [20],[21] found MHR1 to be associated with mitochondrial ribosomal large subunit proteins by high-throughput APMS studies. Overall, this consistent literature support suggests that our classifier makes meaningful predictions. Note that, for this analysis, we selected predictions by using very stringent criteria, requiring that each predicted new member is predicted to be in a co-complexed pair with every member in the MIPS complex. In principle, we could make a larger number of predictions with a more lenient cutoff. A table listing the predicted new members of the MIPS complexes with at least 50% overlap and two overlapping CCPPs is available in the on-line supplement at http://noble.gs.washington.edu/proj/coco.

**Table 2:** Predicted new members of the MIPS complexes.

MIPS ID	Complex	Number	Predicted Members
500.60.10	Mitochondrial ribosomal large subunit	44	MHR1 [49]
440.30.10	mRNA splicing	42	SMB1 and SNU114 [54]
			SYF2, CLF1 and ISY1 [55]
			CUS1 [56]
510.40.20	Kornberg's mediator (SRB) complex	21	SOH1 [57]
160	Exocyst complex	7	EXO84 [58]

The first two columns indicate the MIPS complex, the third column indicates the number of proteins in the complex, and the last column indicates the predicted new members of the complex.

**Figure 7:**
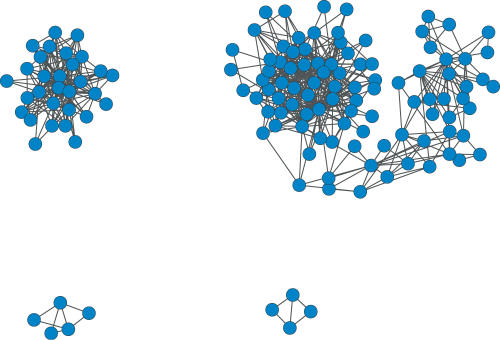
The network of cliques of new CCPPs not in MIPS.

Finally, we analyze the set of novel predictions for which neither protein is a member of a MIPS complex. At an FDR threshold of 10%, this set contains 686 CCPPs among 200 proteins. These predictions can be represented as an undirected graph, with proteins as nodes and predicted co-complex relationships as edges. We identified predicted new complexes as maximal cliques in this graph, where a *clique* is a set of nodes with every pair of nodes in the set connected by an edge, and a *maximal clique* is a clique to which no node in the graph can be added to create a larger clique. In general, finding maximal cliques is an NP-hard problem, but because our network is relatively small and sparse, we were able to perform exhaustive enumeration to identify all maximal cliques. We thereby identified 199 maximal cliques with size of at least 3, including one clique of size 11, one clique of size 10, seven cliques of size 9, and so on down to 48 cliques of size 3. Many of these cliques overlap one another. For example, the clique of size 11 and the clique of size 10 share 9 proteins in common. We therefore created a network consisting of just these predicted cliques. This network, shown in Figure 7, consists of four connected components. We performed GO enrichment analysis on these components with GO::TermFinder [50] and summarized the results in Table 3. All four connected components have significantly enriched GO term annotations for all three ontologies. The members in these four connected components can be found in the on-line supplement http://noble.gs.washington.edu/proj/coco.

**Table 3:** GO enrichment of predicted new complexes not in MIPS.

	Biological Process	Cellular Component	Molecular Function
1	Nuclear mRNA splicing, via spliceosome (9e-52)	Spliceosome (8e-43)	RNA binding (4e-23)
2	Ribosome biogenesis and assembly (4e-50)	Nucleolus (1e-47)	snoRNA binding (1e-07)
3	Protein amino acid glycosylation (1e-06)	Golgi apparatus (7e-07)	Alpha-1,6-mannosyltransferase activity (1e-04)
4	Nucleocytoplasmic transport (7e-06)	Nuclear envelope (3e-06)	Ribonucleoprotein binding (1e-05)

Columns 2, 3, and 4 list the most significantly enriched GO term for each connected component in the predicted network for the biological process, cellular component and molecular function ontologies, respectively. The numbers in the parentheses indicate the corrected p-values of the enrichment calculated with GO::TermFinder.

## Discussion

In this paper, we developed multiple kernels from heterogeneous data sources and combined them in an SVM classifier to predict co-complexed protein pairs. We applied the diffusion kernel to the two-hybrid, APMS and genetic interaction networks, and we found that, in all three cases, a diffusion kernel performs much better than a linear kernel or the mutual clustering coefficient (MCC). A diffusion kernel computes the similarity between two vertices by summing over all paths connecting the two vertices with paths of shorter lengths receiving higher weight. In contrast, a linear kernel or MCC only considers paths of length 2. Our results indicate that taking into account the full network topology improves the prediction of CCPP edges. We also applied our prediction scheme to the protein pairs identified by two recent large scale APMS data sets [21],[22]. Our predicted positives are enriched with protein pairs identified by both groups with high statistical significance, and are consistent with the highly accurate data set of Collins et al. [48] Our method can thus be used to select a subset of these large scale results with better accuracy and reliability.

Different data sources provide complementary information, and each data source may have the best predictive power for a subset of data points. For instance, some protein pairs may have no sequence homologs, and some other protein pairs may not be included in the yeast two-hybrid screen experiments. Therefore, the combination of a variety of data sources has the potential to improve CCPP recognition. Kernel methods present a natural way to combine features by the summation of kernel matrices. Our results show that the TPPK applied to the summation of the sequence and diffusion kernels performs significantly better than either the sequence or the diffusion kernels alone. Inclusion of RBF kernels on five additional data sets improves the ROC_50_ performance further from 0.859 to 0.937. We did not optimize the relative weights of the TPPK and RBF kernels. One future direction is to learn these weights by using semidefinite programming [51], sequential minimal optimization [52] or semi-infinite programming [53].

The method described here is specifically designed to work well in the presence of heterogeneous data—primary sequence, expression, interaction networks, etc. As such, the method can be applied fairly directly to other well-studied eukaryotic genomes. The minimal requirement for applying this method, or indeed any supervised learning algorithm, to a new organism is the availability of data (e.g., protein sequences) and labels (a set of known protein-protein interactions). In practice, the latter is much more difficult to come by. Typically, a genome with a sufficiently large set of high-quality interaction labels will likely also have available non-sequence data such as high-throughput interaction data and expression profiles.
